# Combustion Performance and Thermal Stability of Basalt Fiber-Reinforced Polypropylene Composites

**DOI:** 10.3390/polym11111826

**Published:** 2019-11-06

**Authors:** Chunhong Tang, FengXiang Xu, Guangyao Li

**Affiliations:** 1 State Key Laboratory of Advanced Design and Manufacturing for Vehicle Body, Hunan University, Changsha 410082, China; chun@hnu.edu.cn; 2 Joint Center for Intelligent New Energy Vehicle, Shanghai 200092, China; 3 Hubei Key Laboratory of Advanced Technology of Automotive Components, Wuhan University of Technology, Wuhan 430070, China; xufx@whut.edu.cn; 4 Hubei Collaborative Innovation Center for Automotive Components Technology, Wuhan University of Technology, Wuhan 430070, China

**Keywords:** basalt fiber, polypropylene, thermal stability, combustion, fire hazard

## Abstract

In this study, the thermal stability and combustion performance of basalt fiber reinforced polypropylene (BFRPP) composite and pure polypropylene (PP) were compared. The results show that the basalt fiber has no positive effect on increasing the initial decomposition temperature of PP, but it could reduce the maximum thermal decomposition rate and increase the temperature of the maximum thermal decomposition rate. Adding basalt fiber to PP could slightly reduce the limiting oxygen index. At the same oxygen concentration, the BFRPP burned significantly more slowly than the PP. In addition, during the combustion, it was observed that the BFRPP showed a better anti-melt dripping effect than the PP. The results from the cone calorimeter test show that, under the same external heat flux, the time-to-ignition (TTI) of BFRPP was less than that of PP. This indicated that BFRPP was easier to ignite than PP. It was also found that the reciprocal of the square root of the TTI of both has a linear relationship with external heat flux. BFRPP has a lower peak heat release rate and total heat release than PP. Moreover, BFRPP produced less smoke than PP when burned.

## 1. Introduction

Basalt fiber has good strength and stiffness at high temperature, good acid and alkali chemical corrosion resistance, and its production process is environmentally friendly [1,2]. There has been a recent increase in the use of basalt fiber as a reinforcement in the manufacture of lightweight, low-cost polypropylene (PP) matrix composites for a wide range of applications in the petrochemical, civil engineering and transportation industries [3,4]. For some applications, the threat of fire is inevitable; hence, better fire resistance of materials is necessary because it could reduce casualties and property damage. For example, when buildings or vehicles are exposed to fire, a lower fire hazard index of the material is conducive to people escaping from the scene [5]. Therefore, understanding the combustion characteristics of basalt fiber-reinforced polypropylene (BFRPP) composite is crucial.

Combustion characteristics refer to all physical and chemical changes occurring during the combustion of materials, which can be measured by the limiting oxygen index (LOI), heat release, smoke, mass loss, carbonization and other indicators. The limiting oxygen index (LOI) test, thermogravimetric analysis (TGA) and cone calorimetric test (CCT) are commonly used to evaluate the combustion characteristics of a material.

TGA is often used to evaluate the thermal stability of composites. Fiber-reinforced polypropylene (FRPP) composites typically decompose at temperatures of 300–500 °C, releasing heat and toxic volatiles. The typical TGA curve of FRPP shows that the weight will slowly decrease after heating and then drop sharply in the range of 400–500 °C. Finally, the weight will not drop with the consumption of the reactants, and the slope of the TGA curve is 0 [6].

LOI can be used to compare the fire performance of different materials and indicate how easily the materials burn. Stark et al. [7] compared the LOI value of natural fiber-reinforced composites and polymer matrices. They found that natural fiber-reinforced composites are easier to extinguish in a fire than polymers because natural fiber-reinforced composites require higher oxygen concentrations for sustained combustion. Atabek Savas et al. [8] studied the effect of carbon fiber amounts on the LOI of composites. Their results show that the carbon fiber content increased from 0 to 30 wt. % with the addition of ammonium polyphosphate (APP) at 20 wt. %, and the LOI value of the carbon fiber-reinforced composite decreased from 29.1 to 20.7. They believe that the LOI value of carbon fiber composites decreases with the increase of carbon fiber content, which may be due to the antagonism of carbon fiber and APP. 

CCT is based on the principle of oxygen consumption to assess the flammability of a polymeric material by exposing the sample to a particular radiant flux. It can be used to measure a variety of important flammability characteristics such as the heat release rate (HRR), time-to-ignition (TTI), and rate of smoke release (RSR) when materials are burned [9,10]. CCT can also be used to simulate the combustion characteristics of fiber-reinforced polymer matrix composites under different fire conditions in a laboratory. The heat flux density of the radiation has a significant effect on the combustion characteristics of the composite. Wang et al. [11] studied the effects of different heat fluxes on the combustion characteristics of glass fiber-reinforced polymer (GFRP) composite. The experimental results show that the igniting time of GFRP decreases from 160 to 35 s as the heat flux increases from 25 to 75 kW·m^−2^. The peak of the heat release rate (HRR) curve is advanced, and the peak value increases. As the heat flux increases from 25 to 75 KW m^−2^, the smoke production rate of GFRP is also affected by the external heat flux: the peak value becomes larger and the peak appears longer. Brown and Mathys [12] compared the combustion characteristics of a vinyl ester resin composite of two fiber-reinforced forms of glass, a chopped strand mat and woven cloth. They found that the chopped strand-reinforced composites exhibited lower peak heat release rates than woven fabric-reinforced composites. Atabek Savas, Mutlu, Dike, Tayfun and Dogan [8] studied the flame retardancy of carbon fiber content on the intumescent polypropylene composite. They found that the fiber length had no significant effect on the combustion characteristics of the composite, but an effect for carbon fiber was observed. With the antagonism of ammonium polyphosphate, the oxygen index of the composite decreases as the fiber content increases. Quang Dao et al. [13] studied two different levels of GFRP composites. When the fiber content increased from 56 to 59 vol. %, the critical heat radiant flux decreased from 18 to 14 KW m^−2^, and the thermal response parameter decreased from 435 to 370 kW s^1/2^ m^−2^. At the same time, the increase in the carbon fiber fraction resulted in a decrease in the peak amplitude of the mass loss rate under the same external heat flux. They further proposed a four-stage thermal decomposition mechanism through the analysis of the evolution of mass loss rate. In the first stage, the epoxy resin is cleaved to form a low molecular weight gaseous species and an epoxy-derived compound. In the next two stages, the combustion of the epoxy resin and the liquid monomer solvent was observed, which induced the formation of coke. The final stage is the oxidation of coke and the decomposition of carbon fibers.

To date, the combustion characteristics of BFRPP have not been reported in the literature, and the influence of basalt fiber on the combustion characteristics of polypropylene polymer is still unclear. Hence, the main purpose of this study was to investigate the combustion characteristics of BFRPP and evaluate the effect of basalt fiber on the combustion properties of polymers.

## 2. Materials and Methods 

### 2.1. Materials and Preparation of Composites

**Figure 1:**
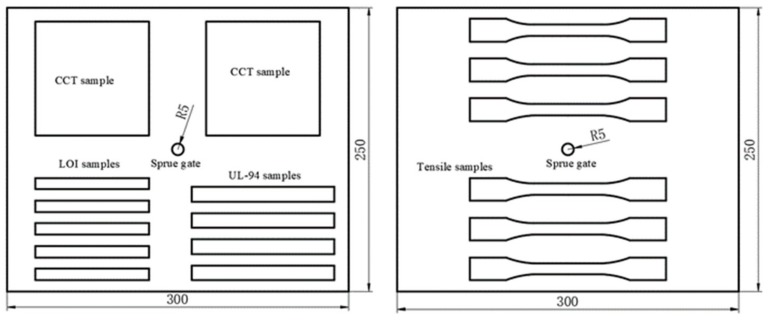
Samples location on the injection molded plates.

The average diameter of continuous basalt fiber was 13 μm. The material properties of basalt fiber are shown in Table 1. The surface of basalt fiber was modified by an amino-silane coupling agent whose main component is γ-aminopropyltriethoxysilane (KH-550). Due to the highly competitive and proprietary nature of commercial fibers, the manufacturer is not able to provide specific formulations of the coupling agent. The PP resin was a block copolymer polypropylene obtained by polymerization of ethylene and propylene. The manufacturing process of BFRPP composite was as follows: firstly, the continuous basalt fiber and PP were made into composite pellets with an average length of about 3 mm by using a twin-screw extruder at temperature of 200 °C, and then these pellets were dried for 24 h at 90 °C. Finally, the pellets were injection molded into a sheet with a size of 250 mm × 300 mm × 4 mm at temperature of 210 °C. The sprue gate is located in the center of the plate, as shown in Figure 1. The fiber content in the BFRPP plate is about 32 wt. %. The average fiber length in the BFRPP plate is about 1 mm. Samples with the corresponding size were cut from the plate for related research (see Figure 1).

### 2.2. Measurement and Characterization

#### 2.2.1. Mechanical Testing

**Figure 2:**
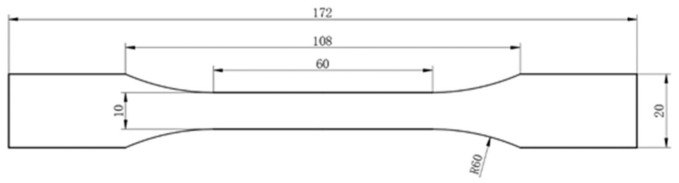
The size of tensile specimen.

The tensile properties were investigated according to ISO 527 standards using an electronic universal testing machine (Model: E45.105-B, MTS Systems Corporation, Oak Ridge, TN, USA). The size of the specimen is shown in Figure 2. The test speed was 5 mm·min^−1^.

#### 2.2.2. Thermogravimetric Analysis (TGA)

The thermogravimetric test environment could be air or inert gas; the thermal decomposition of the former material was accompanied by oxygen, and the latter was used to evaluate the thermal decomposition characteristics of the material itself. The TGA and differential scanning calorimetry (DSC) tests of basalt fiber were carried out in a STA449C TG-DSC analyzer (NETZSCH Ltd., Selb, Germany). About 10 mg of basalt fiber powder was placed in the alumina crucible and tested in an argon (Ar) atmosphere. The flow rate was 40 mL L^−1^, the heating rate was set to 10 °C min^−1^, and the temperature was raised from room temperature to 1000 °C. The TGA tests of BFRPP and PP were also carried out on a STA449C TG-DSC analyzer. The test atmospheres of the two kinds of samples were Ar and air atmospheres, respectively. The flow rate was 40 mL L^−1^, and the heating rate was set to 10 °C min^−1^. To evaluate the thermal decomposition kinetics of BFRPP, thermogravimetric tests of PP and BFRPP composites were also carried out under a nitrogen (N_2_) atmosphere using a TA Q50 (TA Instruments, New Castle, DE, USA) tester with a mass of approximately 10 mg. The sample was placed in a platinum crucible with an N_2_ gas flow rate of 50 mL min^−1^. The heating rates were 10, 20, 30 and 40 °C min^−1^.

The thermal stability of BFRPP and PP could be evaluated by the initial decomposition temperature (*T*_initial_) and the corresponding temperature at which the mass was reduced by 5% (*T*_-5%_), 10% (*T*_−10%_) and 50% (*T*_−50%_). To better analyze the thermal stability of the tested substance, the thermogravimetric (TG) curve could be used to obtain a first-order derivation of the temperature to obtain a differential thermogravimetric (DTG) curve, which could reflect the decomposition of the tested substance at different temperatures. Another important thermal property parameter was the temperature (*T*_p_) corresponding to the maximum mass (weight) loss rate, which was the peak of the first derivative curve of the DTG curve versus temperature [14].

#### 2.2.3. Limiting Oxygen Index (LOI) Testing

The oxygen index of BFRPP and PP was determined according to the ASTM D 2863-2009 standard using a JF-3 Oxygen Index Tester (Chengde Jinjian Testing Instrument Co., Ltd, Chengde, China). The dimensions of the samples were 100 mm × 100 mm × 4 mm. The samples were placed in the 23 °C thermostatic chamber for 24 h before the test. To facilitate the observation of the burning distance, a line, at a distance of 50 mm from the top of the sample, was drawn on the surface of each sample before the test. The LOI was calculated according to Equation (1),

(1) $$\mathrm{LOI}(\%)=C_{f}+kd,$$

where *C_f_* is the final oxygen concentration value; *d* is the difference in oxygen concentration controlled during the test, *d* = 0.2; and *k* is the coefficient obtained by looking up the table in ASTM D 2863-2009 standard.

#### 2.2.4. UL-94 horizontal burning Testing

The horizontal burning tests were carried out on a ZY6017 instrument (Zonsky Instrument CO., LTD., Dongguan, China) on sheet dimension of 125 mm × 13 mm × 4 mm according to UL-94 horizontal standard method. The samples were placed in the 23 °C thermostatic chamber for 48 h before the test. Firstly, lines were drawn at 25 and 100 mm from the end of the sample, and then a 25 mm Bunsen flame was applied to the end of the sample. When the sample burned to the 25 mm mark before 30 s, the Bunsen flame was removed. If the sample constantly burned after displacement of the Bunsen flame, the time for the fire front to travel from the 25 to the 100 mm marks was recorded to calculate the burning rate as follows:

(2) V=L∗60/t,

where *L* is the distance between the two marking lines, *L* = 75 mm; and *t* is the time (measured in minutes) of the flame spread.

#### 2.2.5. Measurement of thermal diffusivity coefficient

The thermal diffusivity coefficients of the BFRPP composite and PP were measured directly by laser-flash method using a LFA467 “Hyper Flash” apparatus (NETZSCH Ltd., Selb, Germany) at temperatures of room temperature. The tested samples were prepared at a size of 10 mm × 10 mm × 1 mm. Since PP is a light-transmitting material, graphite spraying was required on the front and back sides of all samples.

#### 2.2.6. Cone Calorimeter Test (CCT)

The CCT was performed using a FTT0007 cone calorimeter (Fire Testing Technology Ltd., East Grinstead, UK) according to ISO 5660 standard. The sizes of the specimens for CCT were 100 mm × 100 mm × 4 mm. In this study, BFRPP composites and PP specimens were tested under three external radiant heat fluxes of 25, 35 and 50 kW·m^−2^ to evaluate the combustion characteristics of BFRPP under different fire intensities.

## 3. Results and Discussions

### 3.1. Mechanical Properties

**Figure 3:**
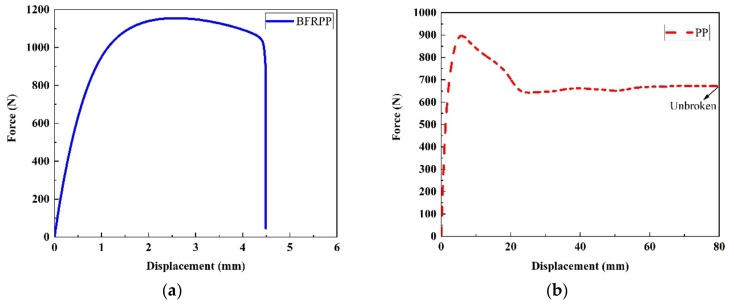
The typical force–displacement curves: (**a**) PP; and (**b**) BFRPP.

The typical force–displacement curves are shown in Figure 3. It can be seen in Figure 3 that the maximum tensile force that the BFRPP specimen can withstand is higher than that of the PP specimen. The tensile strength of the BFRPP sample is 29 MPa, and the tensile strength of the PP sample is 23 MPa. The tensile strength of BFRPP is 26.1% higher than PP. It has been reported in the literature that the KH-550 coupling agent can promote the bonding interface property between PP matrix and basalt fiber [15]. This may be the main reason the tensile strength of BFRPP is higher than PP.

### 3.2. Thermogravimetric Analysis (TGA)

#### 3.2.1. Thermal Stability

**Figure 4:**
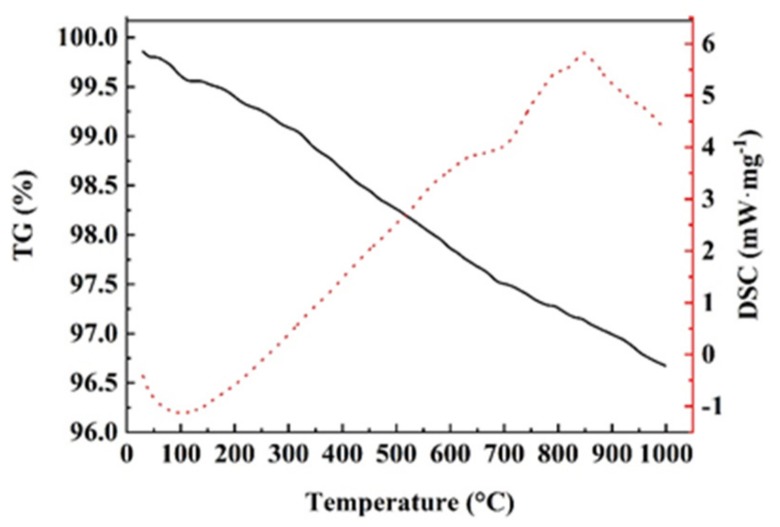
The thermogravimetric (TG) and differential scanning calorimetry (DSC) curves of basalt fiber in an Ar atmosphere.

Figure 4 shows the TG and DSC curves of basalt fiber under an Ar atmosphere. It can be seen from the TG curve of basalt fiber that its thermal stability is superb, and the weight loss is only 3.3% when heated from room temperature to 1000 °C. Within 1000 °C, the mass of the basalt fiber decreases with the increase of temperature, and the weight loss curve is wavy, with multiple peaks. It can also be observed from the DSC curve of basalt fiber that there is a distinct endothermic peak at 849 °C, which is an endothermic reaction. The composition of basalt fiber is mainly aluminosilicate, not a single compound, and its thermal decomposition process includes various reactions such as melting, crystal transformation, sublimation and solid phase transformation [16]. 

**Figure 5:**
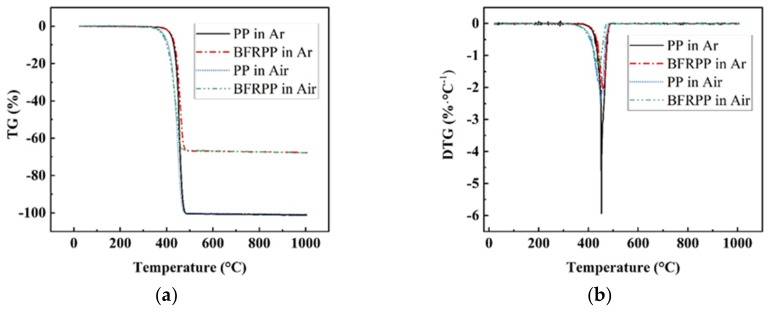
Thermogravimetric curves of samples: (**a**) TG curves; and (**b**) DTG curves.

Figure 5 shows the TG and DTG curves for PP and BFRPP. Some corresponding characteristic data are given in Table 2. In the Ar gas atmosphere, when a certain temperature is reached, a random chain scission occurs in the PP molecular chain. Such random chain scission events occur successively in PP and its degradation products resulting in a molecular weight reduction initially and ultimately a mass loss because the molecular weight of the degraded products become small enough to evaporate [17]. Figure 5 shows that, in the Ar atmosphere, the initial decomposition temperatures of BFRPP and PP are the same, at 403 °C. The *T*_−5%_, *T*_−10%_, and *T*_−50%_ of the PP samples are about 420, 431, and 453 °C. Compared with PP, the addition of basalt fiber increases the *T*_−5%_ (to 423 °C), *T*_−10%_ (to 436 °C) and T_−50%_ (to 463 °C) of PP. This means that the basalt fiber has no positive effect on increasing the initial decomposition temperature of PP, but in the decomposition process, the corresponding temperatures (*T*_−5%_, *T*_−10%_ and *T*_−50%_) of BFRPP are higher than PP under the same mass loss rate. The residual mass percentage of PP at 1000 °C is 0, and that of BFRPP is about 32.28%. The mass percentage of the residue in BFRPP is almost the same as that of the added basalt fiber. Therefore, it can be considered that basalt fiber has no significant effect on increasing the carbonization effect of PP.

In the air atmosphere, the *T*_initial_ of BFRPP is 362 °C, which is lower than PP (369 °C). Both the *T*_initial_ of PP and BFRPP in the air atmosphere are lower than in the Ar gas atmosphere. The *T*_−5%_, *T*_−10%_, and *T*_−50%_ of the PP samples are 390, 407, and 443 °C, while the *T*_−5%_, *T*_−10%_, and *T*_−50%_ of the BFRPP are 385, 401 and 446 °C, respectively. On the one hand, before the decomposition of BFRPP from the beginning to the degradation of 10 wt. %, the corresponding decomposition temperature is lower than PP. On the other hand, when the mass decomposition rate is 50%, the corresponding decomposition temperature is higher than PP. Therefore, it can be concluded that basalt fiber has no positive effect on increasing the initial decomposition temperature of PP, and BFRPP exhibits higher thermal stability than PP only when the decomposition quality reaches a certain level. The reason for this phenomenon is that the initial stage of decomposition, due to the thermal insulation effect of the basalt fiber, is not conducive to the heat conduction to the solid interior of BFRPP sample, leading to a rapid increase in the temperature of the PP on the surface of the BFRPP sample. After the PP on the surface of the BFRPP sample is decomposed, basalt fiber is left. Although the mass change of basalt fiber after heating is small, it absorbs a part of the heat, which causes the thermal decomposition temperature of BFRPP sample to be higher than that of the pure PP sample.

#### 3.2.2. Thermal Decomposition Kinetics

Both Ozawa′s method and Kissinger′s method can be used to study the thermal degradation and decomposition kinetics of polymer composite materials based on the Arrhenius equation [18,19]. The difference is that Kissinger′s method is only applicable to reactions with a reaction mechanism function of *f*(α) = (1 − α)^*n*^. Ozawa′s method does not require assumptions about the reaction mechanism function of the BFRPP composite. These two methods can be mutually verified to ensure the accuracy of the results. Therefore, the activation energy of the thermal decomposition kinetics (*E*_a_) for BFRPP and PP specimens was determined by using Ozawa′s method and Kissinger′s method.

TGA data provided information for the calculation of the *E*_a_ during decomposition. At a linear heating rate, the degree of conversion (α) was defined as

(3) $$\alpha =\frac{m_{0}-m}{m_{0}-m_{f}},$$

where *m*_0_ is the initial mass, *m* is the mass at decomposition temperature, and *m_f_* is the mass at final decomposition reaction.

Based on Arrhenius equation, the *E*_a_ could be determined as follows:

(4) $$\;\frac{d\alpha }{dT}=\;\frac{A}{\beta }e^{\frac{-E_{a}}{RT}}f(\alpha ),$$

where *A* is the pre-exponential factor, *R* is the universal gas constant, *n* is the reaction order, *T* is the temperature, and β is the linear heating rate.

Equation (4) could not be solved explicitly. Ozawa′s method results in *T*_f_ = *T*_0_ + βt (where *T*_f_ is the final temperature; *T*_0_ is the initial temperature; and *t* is time). Substituting Doyle’s approximation into Equation (4), and after a simple conversion, the following expression could be derived:

(5) $$\ln\beta =\;-1.0516\frac{E_{a}}{RT}+const,$$

Therefore, we plotted lnβ as a function of 1/T at a series of β. The *E*_a_ can be calculated from the slopes of the lines according to Equation (6).

(6) $$\;E_{a}=-\mathrm{slope}\frac{R}{1.0516},$$

**Figure 6:**
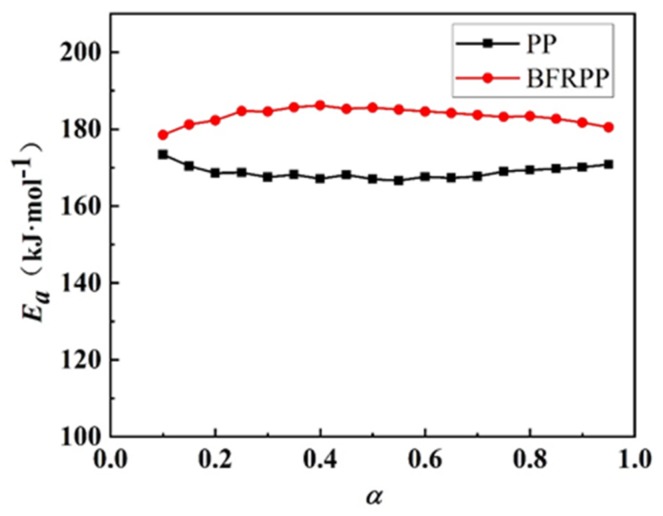
Comparison of activation energy of BFRPP and PP.

The apparent activation energy (*E*_a_) of BFRPP and PP at different conversions α is plotted in Figure 6. It can be seen in Figure 6 that the apparent activation energy of BFRPP was significantly higher than that of PP in the range of α = 0.1–0.95, indicating that the energy required for BFRPP′s thermal decomposition was higher than PP; i.e., BFRPP has higher thermal stability than PP in the range of range of α = 0.1–0.95. The apparent activation energy of BFRPP could be roughly divided into three stages. In the range of α = 0.1–0.4, the apparent activation energy of BFRPP increased with the increase of the degree of conversion. In the range of α = 0.4–0.8, the apparent activation energy remains basically unchanged. In the range of α = 0.8–0.95, the apparent activation energy decreased slightly with the increase of degree of conversion. For PP, when α = 0.1–0.2, the apparent activation energy *E*_a_ decreased as the degree of conversion α increased. At α = 0.2–0.65, the apparent activation energy fluctuated slightly with the increase of the degree of conversion. At α = 0.65–0.95, the apparent activation energy increased with the increase of the degree of conversion.

In the case of Kissinger’s method, the *E*_a_ was described as

(7) $$\ln\left(\frac{\beta }{T_{p}^{2}}\right)=\;-\frac{E_{a}}{RT_{p}}+\ln\frac{AR}{E_{a}},$$

where *T*_p_ is the temperature at which the maximum decomposition occurs at different heating rates. *E*_a_ can be obtained from the slope of the straight-line ln[β/(*T*_p_^2^)] vs. 1/*T*_p_.

The apparent activation energies of PP and BFRPP obtained by Kissinger′s method are shown in Table 3. Using Kissinger′s method, the apparent activation energies of BFRPP and PP in the range of α = 0.1–0.95 were 180 and 162.5 kJ·mol^−1^, respectively. These results are only 1.9% and 3.8% different from the average values of *E*_a_ obtained by Ozawa′s method, respectively.

Thus, we can say that the *E*_a_ values of BFRPP and PP obtained by the two methods are almost the same. Therefore, it could be concluded that, in the range of α = 0.1–0.95, the energy required for thermal decomposition in BFRPP composites was higher than that of PP, and BFRPP has higher thermal stability than PP.

### 3.3. Limiting Oxygen Index (LOI) and UL-94 Horizontal (UL-94 H) Burning Testing

It was found in the LOI test that the BFRPP sample was easier to ignite than the PP sample, which was also affected by the thermal insulation of the basalt fiber. The LOI value of PP and BFRPP samples is shown in Table 4. The LOI value of BFRPP was 18.6, which was lower than the 19.1 of pure PP, indicating that the addition of basalt fiber slightly reduced the LOI value of PP.

**Figure 7:**
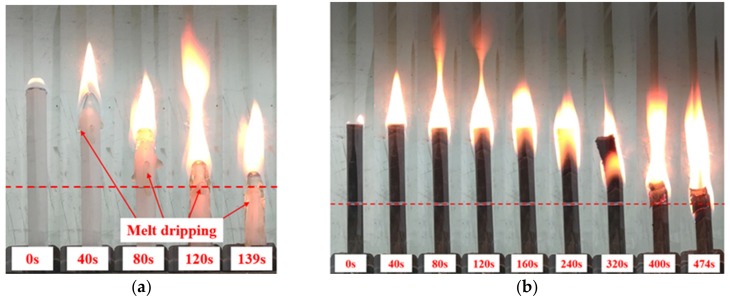
The combustion process of samples at an oxygen concentration of 20%: (**a**) PP; and (**b**) BFRPP.

During the limiting oxygen index test, the combustion process of PP and BFRPP was also analyzed. Figure 7 shows the combustion process of PP and BFRPP samples at 20% oxygen concentration. The distance between the red dotted line and the top of the sample was 50 mm. It can be seen that the burning speed of PP was much faster than BFRPP. It took about 400 s for BFRPP to burn to the marked line. In contrast, it took about 120 s for PP to burn to the marked line. In addition, there was a significant difference in the combustion phenomena between the two samples. The PP sample experienced extremely severe melt dripping during combustion (see Figure 7a). However, no melt dripping of PP was observed in the BFRPP sample. When the PP in the BFRPP sample was burned, the residual basalt fiber was exposed. As the combustion continued, the basalt fiber remaining on the top of the BFRPP sample increased, and finally fell naturally under the action of gravity (see Figure 7b).

**Figure 8:**
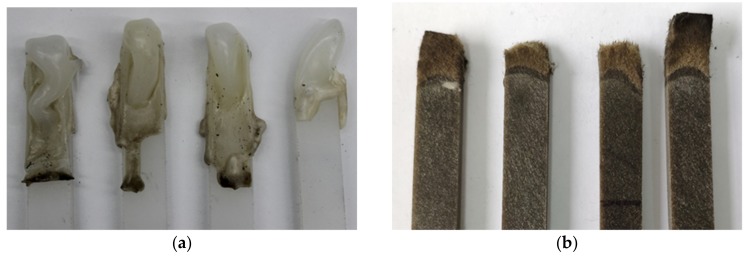
Partial photos of samples after burning: (**a**) PP; and (**b**) BFRPP.

Photographs of some sample residues after the LOI test are shown in Figure 8. Figure 8a shows the PP sample after combustion. It can be seen that the main component of the residue was mainly molten PP, and the surface of the residue was relatively flat and smooth. No residue was left when the PP was completely burned, thus no formation of a carbon layer was observed on the surface of the PP. Figure 8b shows the BFRPP sample after combustion. It can be seen that only the PP in the BFRPP participated in the combustion, and the basalt fiber did not burn.

**Figure 9:**
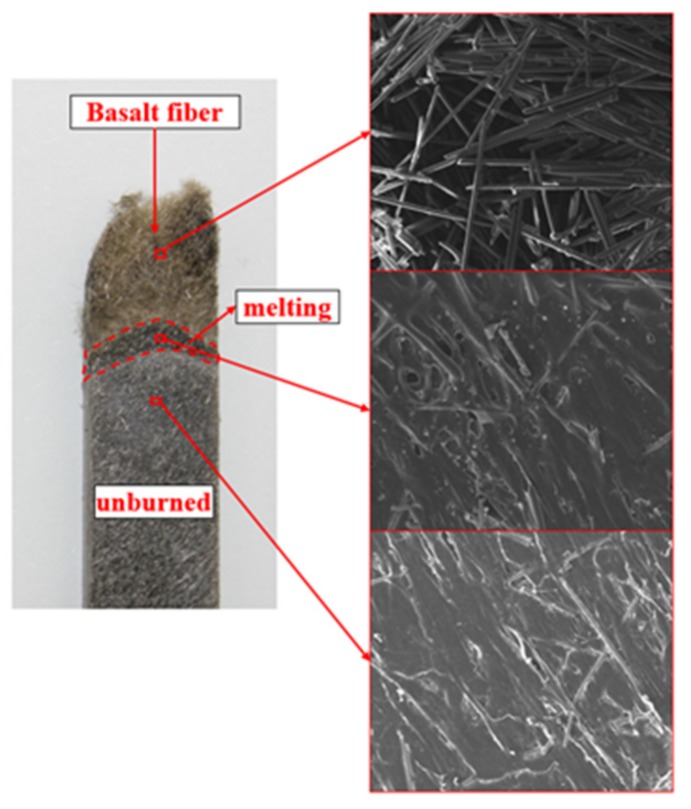
Photos of BFRPP sample after burning.

Figure 9 shows the SEM morphology of the BFRPP after the LOI test. It can be seen that the residue of the BFRPP sample after combustion was basalt fiber, and the basalt fiber maintained its original fiber shape and did not stick together. The BFRPP sample in Figure 9 could be roughly divided into three parts—the basalt fiber zone, the melting zone and the unburned zone—and the boundaries of the three parts were obvious. The PP matrix in the melting zone has a darker color after cooling than the unburned zone. It can be seen from the enlarged view of the melting zone that the combustion of the BFRPP composite has the characteristic of a “candlewick effect”; i.e., the basalt fiber acted as a candlewick and was able to transfer and feedback the pyrolyzed fuel to the flame through capillary action. On the other hand, the BFRPP burned at a slower rate than pure PP. The reason for this was that the basalt fiber isolated a part of the heat and did not accelerate the heat released during the combustion to return to the unburned PP matrix. Therefore, the thermal decomposition rate of the PP in the BFRPP sample was slower than that of the PP sample, resulting in a longer burning time and better anti-melting dripping of the BFRPP composite.

The results of UL-94 horizontal burning test for PP and BFRPP are listed in Table 5. Table 5 shows that the average burning rate of PP and BFRPP samples did not exceed 40 mm·min^−1^. However, one PP sample has a burning rate of 40.9 exceeding 40 mm·min^−1^. Hence, the BFRPP can achieve a classification of HB, while PP cannot.

**Figure 10:**
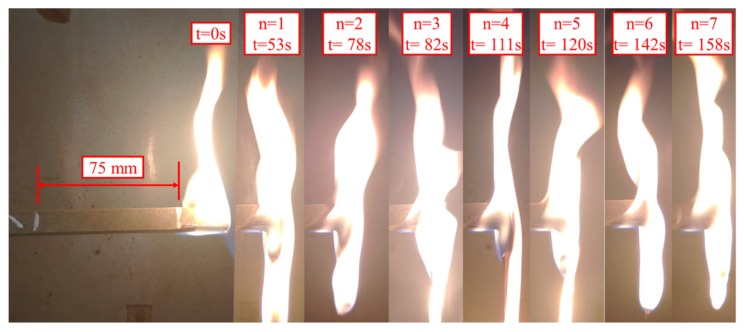
The combustion process of BFRPP sample during UL-94 horizontal burning test.

**Figure 11:**
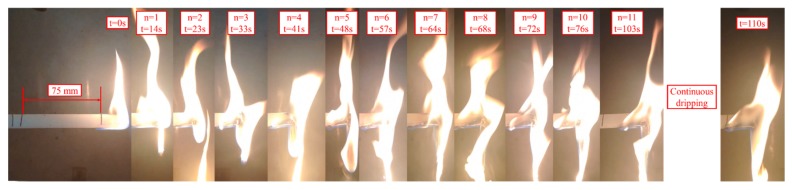
The combustion process of PP sample during UL-94 horizontal burning test.

To analyze the melt dripping of horizontal burning, we recorded the combustion process of the BFRPP and PP sample with a camera. The flame spreads downward during the combustion of the LOI test, while the flame spreads horizontally during the ul-94 horizontal burning test. Figure 10 and Figure 11 show the combustion process of the BFRPP sample and the PP sample during UL-94 horizontal burning test, respectively. In the two figures, “n” represents the number of droplets and “t” represents time. It can be seen that both the BFRPP sample and the PP sample have a significant melt dropping phenomenon. However, the PP sample has a shorter time to first dripping than the BFRPP sample. The PP sample showed a first dripping only when it burned for 14 s, while the BFRPP sample showed the first dripping when it burned for 53 s. On the frequency of dripping, the PP sample had a greater number of drippings than the BFRPP sample. As shown in Figure 10, seven melt drippings occurred in the BFRPP sample burning for a length of 75 mm. In Figure 11, more than 12 melt drippings occurred in the PP sample burning for a length of 75 mm. The PP sample showed continuous dripping after burning for 103 s.

### 3.4. Cone calorimeter test (CCT)

#### 3.4.1. Time-to-ignition (TTI)

The TTI values of PP and BFRPP at different radiant intensities are shown in Table 6. It can be seen that the higher was the heat flux, the easier were the PP and BFRPP to ignite. As the heat flux increased from 25 to 50 kW·m^−2^, their ignition time decreased from approximately 120 s to approximately 40 s: a reduction of nearly two times. BFRPP composites have smaller TTI values than PP, and the higher was the heat flux, the more distinct was the difference. At 25 kW·m^−2^ radiant intensity, the ignition time of BFRPP was only 1 s shorter than that of PP, but, at a higher heat flux of 35 and 55 kW·m^−2^, the ignition time of BFRPP was higher than that of PP.

**Figure 12:**
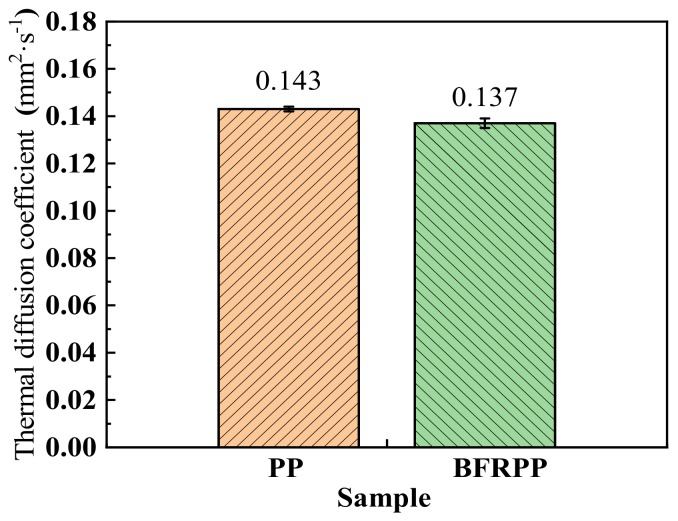
The thermal diffusivity of the PP and BFRPP.

To find out why the TTI of BFRPP is smaller than the TTI of PP, we measured their thermal diffusivity and the results are shown in Figure 12. It can be seen that the thermal diffusivity of the BFRPP composite is smaller than that of PP. When the material was subjected to an external heat source, it took a certain amount of time to collect enough heat to achieve ignition point. The thermal diffusivity of the BFRPP composite is smaller than that of PP, which was not conducive to the conduction of heat into the solid interior of BFRPP composite. When the BFRPP was subjected to an external heat source, the temperature of the PP on the surface of the BFRPP close to the external heat flux rose more quickly than that of pure PP. The PP on the BFRPP sample can reach the ignition point in a shorter time.

In addition, it was found that there was a linear relationship between the reciprocal of the square root of TTI and heat flux. The fitting formulas for obtaining PP and BFRPP were as follows:

PP:

(8) y = 0.00259I+0.02716

BFRPP:

(9) y = 0.00298I+0.01895

where *I* is the heat flux, and *y* is $1/\sqrt{\mathrm{TTI}}$.

**Figure 13:**
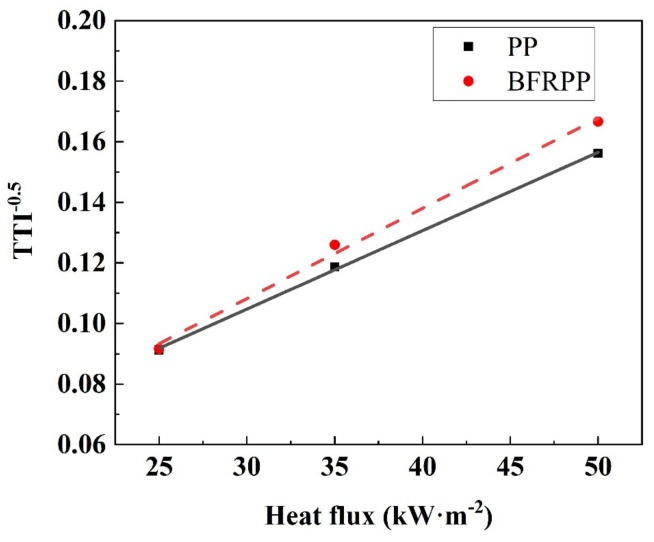
The relationship between the reciprocal of square root of TTI and heat flux.

Figure 13 shows the regularity of the reciprocal of the square root of TTI as a function of the external heat flux. It can be seen that there was a good linear relationship between them.

#### 3.4.2. Heat Release Rate (HRR) and Total Heat Release (THR)

**Figure 14:**
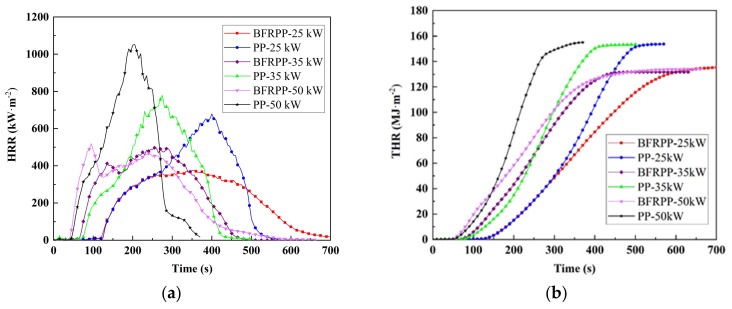
Heat release rate (HRR) and total heat release (THR) curves: (**a**) HRR and (**b**) THR.

The HRR curves for PP and BFRPP are shown in Figure 14a, and the corresponding THR curves are shown in Figure 14b. It can be seen that, as the external heat flux increased, the width of the HRR curve of PP was significantly narrower, indicating that its sustained burning time was reduced. The widths of the HRR curve of BFRPP at 35 and 50 kW·m^−2^ were close, and both were significantly narrower than at 25 kW·m^−2^. This indicates that, to some extent, the increase in heat flux would shorten the duration of BFRPP’s continuous combustion, but after a certain threshold, the heat flux has little effect on the continuous burning time of BFRPP. Additionally, under the radiant heat flux of 35 and 50 kW·m^−2^, the HRR curves of BFRPP show a multi-peak phenomenon The HRR curves has only one secondary peak before the maximum peak. The multi-peak phenomenon may be related to char formation. However, no char residual was observed in the residue photographs of BFRPP sample at 50 kW·m^−2^ heat fluxes (see Figure 17). Thus, it is believed that the multi-peak phenomenon is caused by the fact that the basalt fiber begins to hinder heat feedback after the PP on the surface of BFRPP is completely burned. When the BFRPP under the higher external heat fluxes (35 and 50 kW·m^−2^), the PP on the surface of BFRPP is violently burned, and a large amount of heat is released in a short time. When the PP on the surface of the BFRPP is completely burned, a layer of basalt fiber is interspersed between the flame and the unburned area. At this moment, the heat cannot be quickly and largely fed back to the unburned area, thus the pp of the unburned area is difficult to decompose in large quantities for a short period of time to provide a large amount of fuel for continuous combustion.

The fire propagation index (FPI) and the fire growth index (FGI) could be calculated from the results of CCT. The FPI and FGI were defined as follows [20]:

(10) $$\mathrm{FPI}=\frac{\mathrm{TTI}}{\mathrm{pHRR}},$$

(11) $$\mathrm{FGI}=\frac{\mathrm{pHRR}}{t_{p}},$$

where pHRR is the peak of the HRR and *t*_p_ is the time corresponding to the maximum HRR. The higher is the FPI value, the stronger is the flame-retardant properties of the material and the is lower the fire risk. The larger is the FGI value, the more likely is the fire to spread and expand, and the greater is the risk of fire. The corresponding FPI and FGI results are listed in Table 7.

Table 7 shows that the heat flux has a significant effect on the FPI and FGI of PP and BFRP materials. As the heat flux increased, the FPI of both PP and BFRPP decreased, and the FGI increased. The FPI value of BFRPP was higher than that of pure PP, which was about 1.4–1.8 times that of pure PP.

At 25 and 35 kW·m^−2^, the FGI of BFRPP was only 62%–69% of pure PP. This shows that, at lower radiant intensity, basalt fiber could inhibit the spread of fire well during pure PP combustion. At a heat flux of 50 kW·m^−2^, the FGI value of BFRPP was comparable to that of pure PP, which was about 107% of pure PP. This shows that, under the higher heat flux, due to the increase of BFRPP combustion intensity, the heat released in a short time was extreme, which weakened the effect of basalt fiber of preventing flame spread.

#### 3.4.3. Rate of Smoke Release (RSR) and Total Smoke Release (TSR)

The smoke generated during combustion was considered to be an important factor in the death of people through asphyxiation and/or the inhalation of toxic gases [21,22]. Therefore, it is very important to study the smoke suppression of BFRPP composites.

**Figure 15:**
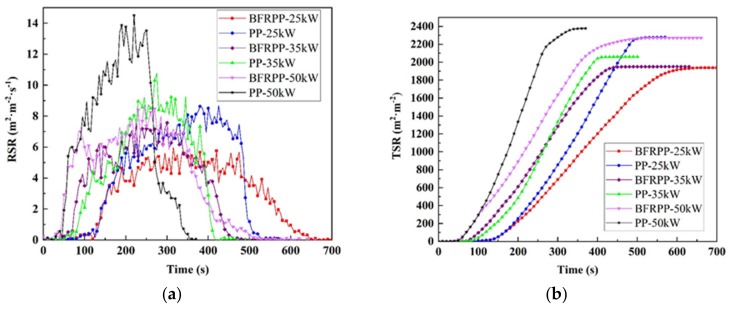
Rate of smoke release (RSR) and total smoke release (TSR) curves: (**a**) RSR and (**b**) TSR.

The RSR curves for PP and BFRPP are shown in Figure 15a. The corresponding characteristic parameter values are listed in Table 8. The peak rate of smoke release (pk-RSR) of PP was 8.68 m^2^·m^−2^·s^−1^ at 25 kW·m^−2^. When the heat flux was increased to 35 and 50 kW·m^−2^, the corresponding pk-RSR increased to 10.77 and 14.50 m^2^·m^−2^·s^−1^, respectively. The phenomenon that pk-RSR increased with increasing external heat flux was also observed in BFRPP. Under the conditions of heat flux of 25, 35 and 50 kW·m^−2^, the pk-RSR of BFRPP was 5.98, 7.61 and 8.74 m^2^·m^−2^·s^−1^, respectively. This was 31.1%, 29.3% and 39.7% lower than pure PP, respectively. Under these three different heat fluxes, the av-RSR of BFRPP was 2.33, 3.05 and 3.39 m^2^·m^−2^·s^−1^, and that of PP was 3.97, 4.04 and 6.34 m^2^·m^−2^·s^−1^, respectively It can be seen that the av-RSR of BFRPP was also significantly lower than that of pure PP, which was 41.3%, 24.5% and 46.5% lower than that of PP, respectively.

Figure 15b shows the curves of TSR as a function of time. The total amount of smoke produced did not decrease significantly with increasing heat flux. The PP sample has the best combustion state at 35 kw·m^−2^ and produces the least amount of smoke, which was 2061 m^2^·m^−2^. BFRPP produces the most smoke at a heat flux of 50 kW·m^−2^, which was 2271 m^2^·m^−2^. As the heat flux increased from 25 to 50 kW·m^−2^, the TSR of BFRPP was 1938, 1951 and 2271 m^2^·m^−2^, which was 15.0%, 5.3% and 4.5% lower than pure PP, respectively. In summary, BFRPP has a lower pk-RSR and TSR than PP at the same heat flux. BFRPP produced less smoke than PP when burned. Therefore, the fire hazard of BFRPP was lower than PP.

#### 3.4.4. Total Mass Loss (TML)

The TML is also an important parameter for evaluating combustion performance and can reflect the carbonization effect of materials in CCT tests [23].

**Figure 16:**
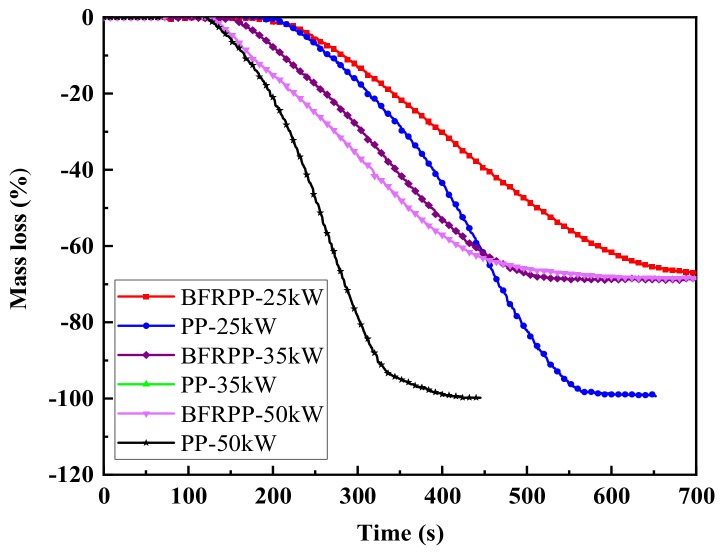
Total mass loss curves.

Figure 16 shows the mass loss versus time curve for PP and BFRPP. It can be seen that the heat flux increased from 25 to 50 kW·m^−2^, and the TML of PP and BFRPP remained basically unchanged. This indicates that the heat flux has no effect on the TML of PP and BFRPP. The TML of BFRPP is 68.3%–68.6%, and the residual mass after combustion is 31.7%–31.4%, which is equivalent to the mass fraction of added basalt fiber. This also shows from another angle that basalt fiber has no obvious effect on promoting pure PP into char. In contrast, pure PP has a TML of 98.6%–99.7%, which is almost completely burned and has no residue. The reason it did not reach 100% was because there was a very small part of PP remaining on the corners of the foil paper when the flame was extinguished.

#### 3.4.5. Residue Analysis

**Figure 17:**
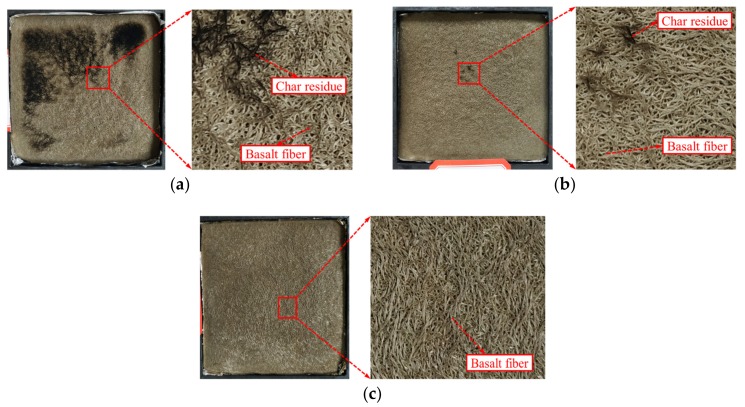
Photos of BFRPP sample after cone calorimeter test (CCT): (**a**) 25 kW·m^−2^; (**b**) 35 kW·m^−2^; and (**c**) 50 kW·m^−2^.

Figure 17 shows a photograph of the residue of the BFRPP sample after CCT. It can be seen that, under the radiant intensity of 35 and 50 kWm^−2^, the residues of the BFRPP composite after combustion are all basalt fibers with almost no PP resin. Under the radiant intensity of 25 kW·m^−2^, there is a small amount of pyro-black residue (char residue) on the surface after BFRPP combustion. This may be because the smoke generated by combustion is smaller under this radiant intensity, and a small amount of charcoal does not follow the smoke. The air is removed and left in the residue. The combustion of BFRPP composites is only the PP in the material involved in the combustion. When the PP is burned, a layer of basalt fiber is formed. Due to the thermal insulation effect of basalt fiber, the basalt fiber layer can slow down the decomposition of PP, reduce mass transfer and heat transfer, and reduce the heat release rate and smoke release rate of BFRPP. In addition, BFRPP’s thermal decomposition activation energy (with a degree of conversion greater than 10%) is also higher than PP, thus the BFRPP composite material has a slower burning rate than PP and a smaller peak heat release rate.

## 4. Conclusions

In this study, the thermal stability and combustion performance of basalt fiber reinforced polypropylene (BFRPP) composite and pure polypropylene (PP) were investigated. The main conclusions could be drawn as follows:

1. The limiting oxygen index (LOI) test showed that the LOI value of BFRPP was 18.6, which was lower than the 19.1 of pure PP. At the same oxygen concentration, it took more time for the BFRPP composite to burn for the same distance as the PP. Additionally, during the combustion, the BFRPP sample showed a better anti-dropping effect than the PP sample.
2. Thermogravimetric analysis (TGA) showed that basalt fiber had no positive effect on increasing the initial decomposition temperature of PP. The initial decomposition temperature of BFRPP and PP was the same in the Ar gas atmosphere. In an air atmosphere, the initial decomposition temperature of BFRPP was slightly lower than PP. Whether in an Ar gas or air atmosphere, the temperature of the maximum thermal decomposition rate of BFRPP was lower than PP.
3. Using Kissinger’s method, the apparent activation energies (Ea) of BFRPP and PP in the range of 0.1–0.95 degrees of conversion were 180 and 162.5 kJ·mol−1, respectively. The results and the average values of Ea obtained by Ozawa′s method differed only by 1.9% and 3.8%, respectively. That means that the Ea values of BFRPP and PP obtained by the two methods were almost the same. Therefore, it could be concluded that, in the range of 0.1–0.95 degrees of conversion, the energy required for thermal decomposition in BFRPP composites was higher than that of PP. The BFRPP had higher thermal stability than PP.
4. Results from cone calorimeter test (CCT) show that the heat release rate (HRR), total heat release (THR), rate of smoke release (RSR) and total smoke release (TSR) of BFRPP composites were lower than PP. As the heat flux increased, the fire propagation index (FPI) of both PP and BFRPP decreased, and the fire growth index (FGI) increased. The FPI value of BFRPP was higher than that of PP, which was about 1.4–1.8 times that of PP. This indicated that basalt fiber has a good effect on reducing the fire risk and fire propagation speed of PP. The higher is the thermal heat flux, the lower are the time-to-ignition (TTI) values of the PP and BFRPP, but the total heat of release of PP and BFRPP was independent of the external heat flux.

## Figures and Tables

**Table 1:** Material properties of basalt fiber and polypropylene.

Materials	Tensile (Yield) Strength (MPa)	Tensile Modulus (GPa)	Density (g·cm^−3^)
Basalt fiber	1500 ± 300	80 ± 5	2.7 ± 0.1

**Table 2:** Thermal decomposition characteristic parameters of PP and BFRPP samples.

Condition	Sample	*T*_initial_ (°C)	*T*_−5%_ (°C)	*T*_−10%_ (°C)	*T*_−50%_ (°C)	*T*_P_ (°C)	Residue (%)
Ar	BFRPP	403	423	436	463	459	32.38
	PP	403	420	431	453	453	0
Air	BFRPP	362	385	401	446	444	32.07
	PP	369	390	407	443	448	0

**Table 3:** Apparent activation energy (*E*_a_) of BFRPP and PP obtained by Kissinger′s method.

Sample	*E*_a_ (kJ·mol^−1^)
BFRPP	180.0
PP	162.5

**Table 4:** LOI and UL-94 test for basalt fiber and polypropylene.

Materials	LOI (MPa)
Polypropylene	19.1 ± 0.1
BFRPP	18.6 ± 0.1

**Table 5:** Results of UL-94 horizontal burning test for PP and BFRPP.

Samples	Average Burning Rate (mm·min^−1^)	Maximum Burning Rate (mm·min^−1^)	Classification *	Remarks
PP	33.1 ± 5.9	40.9	Unclassified	Burning with drips
BFRPP	25.1 ± 2.5	28.5	HB	Burning with drips

* Classification HB: The samples, with thickness of 3.0–13 mm, may not have a burning rate exceeding 40 mm·min^−1^ over a 75 mm span.

**Table 6:** Time-to-ignition (TTI) of PP and BFRPP samples under different heat fluxes.

Intensity (kW·m^−2^)	TTI (s)	
	PP	BFRPP
25	120	119
35	71	63
50	41	36

**Table 7:** The fire propagation index (FPI) and the fire growth index (FGI) of PP and BFRPP samples under different heat fluxes.

Intensity (kW·m^−2^)	FPI		FGI	
	PP	BFRPP	PP	BFRPP
25	0.18	0.32	1.70	1.06
35	0.09	0.13	2.83	1.95
50	0.04	0.07	5.13	5.47

**Table 8:** Peak rate of smoke release (pk-RSR) and average rate of smoke release (av-RSR).

Intensity (kW·m^−2^)	pk-RSR (m^2^·m^−2^·s^−1^)		av-RSR (m^2^·m^−2^·s^−1^)	
	PP	BFRPP	PP	BFRPP
25	8.68	5.98	3.97	2.33
35	10.77	7.61	4.04	3.05
50	14.50	8.74	6.34	3.39
